# Open adjacencies and *k*-breaks: detecting simultaneous rearrangements in cancer genomes

**DOI:** 10.1186/1471-2164-15-S6-S4

**Published:** 2014-10-17

**Authors:** Caleb Weinreb, Layla Oesper, Benjamin J Raphael

**Affiliations:** 1 Center for Computational Molecular Biology, Brown University, Providence, RI, USA; 2 Department of Computer Science, Brown University, Providence, RI, USA

**Keywords:** chromothripsis, chromoplexy, cancer, genome rearrangements, DNA sequencing

## Abstract

**Background:**

The evolution of a cancer genome has traditionally been described as a sequential accumulation of mutations - including chromosomal rearrangements - over a period of time. Recent research suggests, however, that numerous rearrangements may be acquired *simultaneously *during a single cataclysmic event, leading to the proposal of new mechanisms of rearrangement such as chromothripsis and chromoplexy.

**Results:**

We introduce two measures, *open adjacency rate *(OAR) and *copy-number asymmetry enrichment *(CAE), that assess the prevalence of simultaneously formed breakpoints, or *k*-breaks with *k >*2, compared to the sequential accumulation of standard rearrangements, or 2-breaks. We apply the OAR and the CAE to genome sequencing data from 121 cancer genomes from two different studies.

**Conclusions:**

We find that the OAR and CAE correlate well with previous analyses of chromothripsis/chromoplexy but make differing predictions on a small subset of genomes. These results lend support to the existence of simultaneous rearrangements, but also demonstrate the difficulty of characterizing such rearrangements using different criterion.

## Introduction

Cancer is driven by somatic mutations in a population of cells [1]. These somatic mutations range in scale from single nucleotide mutations to large-scale chromosomal rearrangements. Traditionally, the evolution of a cancer genome has been described as a sequential accumulation of such mutations over many cell divisions. In 2011, however, Stephens *et al*. [2] suggested that cancer genomes may also acquire tens to hundreds of genomic rearrangements simultaneously as part of a one-time catastrophic event termed *chromothripsis*. This hypothesis was formed as a means of describing observations in data that seemingly could not be described using the standard sequential model of genome rerragements. A related phenomenon reported by Berger *et al*. [3] was later named *chromoplexy *by Baca *et al*. [4]. Both chromothripsis and chromoplexy involve simultaneous breakage and repair at multiple genomic locations, although with slight differences: e.g. chromoplexy is proposed to favor inter-chromosomal over intra-chromosomal rearrangements.

Simultaneous breakage and repair at multiple genomic locations has not yet been measured *in vivo*. Thus, to infer that such an event has occurred one must argue that simultaneous rearrangement is a more plausible explanation for the observed sequencing data than sequential accumulation of rearrangements. Several different signatures have been proposed as the defining characteristics of chromothripsis [5,6] including clustering of rearrangement breakpoints and a small number of oscillating copy number states. While these signatures are suggestive of a simultaneous, or *one-off*, rearrangement event, they do not conclusively establish the occurrence of such an event. In addition, there is variability in how these criteria are implemented [2,7,8] making it unclear how to interpret or compare results across different studies.

The lack of formal models and definitions for detecting chromothripsis and chromoplexy has led to a growing debate about whether these are true phenomena [9,10]. For instance, Sorzano *et al*. [9] suggest that the observed clustered rearrangement breakpoints do not exist in every cell, but rather reflect heterogeneity in the tumor population as a result of an event such as breakage-fusion-bridge (B/F/B) cycle. The fundamental question underlying this debate is how to identify *simultaneous *acquisition of rearrangements - the defining feature of chromothripsis/chromoplexy - in a cancer genome, given sequence data from a tumor sample and matched normal.

The original chromothripsis publication [2] used Monte Carlo simulations to demonstrate that it was unlikely to observe only a few copy number states under a sequential model. While variations on this approach have been adopted in several other studies [7,8], recent reports have questioned the conclusions drawn from this approach. For example, [10] demonstrate that a small but significant proportion (3.9%) of simulated datasets with sequential accumulation of 50 *− *55 breakpoints exhibit three or fewer copy states, thus showing a high false positive rate with this approach. Recently, other methods for identifying simultaneously formed rearrangement clusters have been proposed. ShatterProof [11] provides a framework for combining the various proposed criteria of chromothripsis [5] to generate a composite likelihood score. ChainFinder [4] detects chromoplexy using a graph based model which identifies closed chains of rearrangements that are unlikely to have arisen independently.

Here we introduce two measures of chromothripsis/chromoplexy based on the properties of the adjacencies and copy number changes that are measured by high-throughput sequencing. Since the defining characteristic of chromothripsis/chromoplexy is the simultaneity of breakpoint formation, we define the *open adjacency rate *(OAR) and *copy-number asymmetry enrichment *(CAE) in order to assess the prevalence of simultaneously formed breakpoints. In terms of the models introduced in the genome rearrangement community, genome rearrangements can be modeled as double cut and join (DCJ) operations, where two double-stranded breaks (DSBs) are introduced and repaired in an aberrant configuration [12]. Simultaneous breakage and repair at multiple sites is an operation with more than two cuts, and can be modeled as a *k*-break [13]. We note that in general, a *k*-break may be equivalent to a sequence of DCJ operations. However, under certain conditions described below an observed *k*-break with *k >*2 cannot be equivalently described by a sequence of DCJ operations. Thus, chromothripsis/chromoplexy is the occurrence of one or more *k*-breaks with *k >*2. The OAR and the CAE use different data as input, but both aim to provide an estimate in answer to the following question: given a genome, what proportion of the observed breakpoints were formed in *k*-breaks with *k >*2?

We compute the OAR and CAE on 121 cancer genomes from two datasets that were previously screened for chromothripsis/chromoplexy [7,4]. We find that both measures correlate well with the predicted classifications of chromothripsis/chromoplexy versus sequential (*p <*10^*−*3 ^on data from [7] and *r *= 0.73 on data from [4]), but differ on a small subset of genomes. Visual inspection of the genomes for which OAR makes differing predictions suggest that they have been mis-classified in the published analyses.

## Methods

### Definitions and preliminaries

We consider a *derivative genome *to be a genome that is formed from the normal, or *reference genome *through a series of *k*-breaks. A *k*-break is an operation that cuts the genome at *k *locations and joins the resulting free ends together [13]. *k*-breaks are a general purpose model for structural variation in cancer, since they formally describe a diverse set of rearrangement types including balanced rearrangements such as translocations, inversions and transpositions as well as deletions.

**Figure 1:**
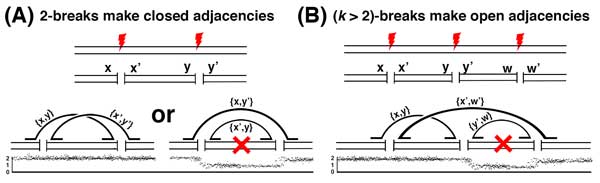
**Examples showing a 2-break and 3-break**. **(A) **In a 2-break, two breaks produce four breakends, organized into counterpart pairs *x, x′ *and *y, y′*, Aberrant repair leads to an inversion/translocation (left) with adjacencies {*x, y*} and {*x′, y′*} or a closed loop that is then lost resulting in a deletion (red X, right). In both cases, all adjacencies are closed. This can be detected as counterpart-symmetry for the inversion adjacencies ({*x, y*}, {*x′, y′*}, left) and copy-number symmetry for the deletion adjacency ({*x, y′*}, right), since due to copy number loss Δ(*x*) = Δ(*y′*) = *−*1. **(B) **In a (*k >*2)-break, *k *breaks produce 2*k *breakends which are aberrantly repaired. Closed loops formed in this process can result in deletions (red X). The resulting adjacencies are open, since for each adjacency *A *the counterparts of the two breakends in *A *are not themselves adjacent. For example, *x *and *y *are adjacent but *x′ *and *y′ *are not. This can be detected using counterpart-asymmetry (e.g. {*x, y*}, since *x′ *is adjacent to *w′ *but *w′ ≠ y′*) or copy-number asymmetry (e.g. {*x′, w′*}, since Δ(*x′*) = 0 while Δ(*w′*) = *−*1).

Formally, we define a *breakend *to be an oriented position on the genome, representing one side of a break (e.g. *x *= (chr17:105227, +)). Thus, each *k*-break produces 2*k *breakends, which are then joined together in an aberrant configuration in the derivative genome. Note that 2-breaks are equivalent to double cut and join (DCJ) operations [12]. Depending on how the resulting breakends are joined, a 2-break models either a translocation, an inversion, or creates a new circular chromosome (Figure 1A). In the last case, if the breakends are on the same chromosome and this circular chromosome is lost, the result is a deletion of the intervening segment. Pairs of breakends that were separate before the breakage but connected after the repair (i.e. in the derivative genome) are called *adjacent*. An unordered pair *A *= {*x, y*} of adjacent breakends is called an *adjacency*. Adjacencies are the signal left by *k*-breaks in the derivative genome. Pairs of breakends connected before the breakage (i.e. in the reference genome) are called *counterparts*. We denote counterpart breakends using a prime, so that if *x *is a breakend, *x′ *is its counterpart. For example, a break occurring between nucleotides *n *and *n *+ 1 will generate counterpart breakends *x *= (*n*, +) and *x′ *= (*n *+ 1*, −*).

### Modeling cancer genomes with *k*-breaks

We model the process of genome rearrangements in cancer as follows. Each tumor begins as a non-mutated founder cell containing the reference genome. Over time, a sequence of *k*-breaks occur in the founder cell's lineage, eventually forming the derivative genome which is revealed at the time of sequencing. *k*-breaks occur according to two assumptions:

1 There is no breakpoint reuse; i.e. breaks never occur in the same location twice.

2 All breakends are fused; i.e. no new telomeres are formed. Note, the formation of new closed loops of DNA is allowed

The "no breakpoint reuse" assumption is a subtle issue in evolutionary comparisons [14,15] where the breakends of genome rearrangements are determined as boundaries of synteny blocks from sequence alignments. These boundaries may be ambiguous due to subsequent mutations and/or repetitive sequences at the boundaries, leading to the identification of *breakpoint regions *rather than precise breakends. This lack of resolution is less of an issue in cancer data from high-throughput sequencing where we expect that any breakpoint that is detected is also localized precisely (within a few hundred nucleotides), as there has been little time for subsequent mutations to obscure this breakpoint.

### Open and closed adjacencies

Let  A be the set of all adjacencies produced by a sequence of *k*-breaks that transform the reference genome into a derivative genome.  A should be thought of as a complete 'record' of all the somatic rearrangements that occurred, and not only those that can be measured in the derivative genome; i.e.  A contains adjacencies that may be removed by subsequent deletions in the creation of the derivative genome. Chromothripsis and chromoplexy are putative rearrangement mechanisms in which many breaks occur simultaneously followed by aberrant repair of the resulting breakends, and thus is modeled as the occurrence of one or more *k*-breaks with large *k*. Under the "no breakpoint reuse" and "all breakends fused" assumptions listed above, the occurrence of a *k*-break with *k >*2 will leave a specific signature in the set  A.

Let *A *∈  A be an adjacency with breakends *x *and *y*. From *x*, we infer that at some time a DNA break occurred at *x*'s location. This break would have produced an additional breakend *x′*, the counterpart of *x*. Similarly the break at *y *would have generated a counterpart breakend *y′*. Since adjacencies (hence breakends) are never removed from  A, both *x′ *and *y′ *can be found in adjacencies *B, C ∈ * A. We now ask, when does *B *= *C*? The answer depends on *k*. If *A *was produced by a 2-break, then no other breakends would have been present at the time, forcing *x′ *and *y′ *to form an adjacency (Figure 1A). On the other hand, if *k >*2, then additional breakends would have been available for fusion with *x′ *and *y′ *(Figure 1B). To distinguish between these scenarios we make the following definition.

**Definition 1 ***Given the set * A*of adjacencies produced by a sequence of k-breaks, A *= {*x, y*} *∈ * A*is *closed *if *{*x′, y′*} *∈ * A*; otherwise A is *open.

Every *k*-break generates *k *adjacencies. When *k *= 2, these adjacencies must be closed. Conversely, every open adjacency must have come from a *k*-break with *k >*2. For a given adjacency set  A, let  A^2 ^the subset of adjacencies produced by 2-breaks and let  A*^k^* be the subset produced by (*k >*2)-breaks, so that  A =  A^2 ^*∪ * A*^k^*. Let O( A) be the set of open adjacencies in  A. We have the following.

**Observation 1 ***For every adjacency set * A, O( A) *⊂ * A*^k^*.

### Two signatures of open adjacencies

Our goal is to detect chromothripsis/chromoplexy by inferring the history of *k*-breaks that gave rise to an observed set of adjacencies and copy number aberrations. In particular, we are interested deriving a lower bound for the number of adjacencies produced in *k*-breaks with *k >*2. As described above, this can be accomplished by counting open adjacencies. However, "open" and "closed" are theoretical categories, describing the etiology of an adjacency, rather than its structure in the derivative genome. In particular, subsequent rearrangements or experimental error may obscure whether adjacencies are open or closed. Thus we need to define signatures of open adjacencies that can be robustly applied to real data. We define two such signatures below: (1) *counterpart-asymmetry *; and (2) *copy-number asymmetry*.

Let  A be the complete set of adjacencies produced by a sequence of *k*-breaks and $\tilde{A}$*⊆ * A be the subset observed in genome sequencing data from the derivative genome. Consider an adjacency *A *= {*x, y*} *∈ *$\tilde{A}$. If *A *is open then the counterpart breakends *x′ *and *y′ *must belong to separate adjacencies in  A, say {*x′, w*} and {*y′, z*} where *w ≠ y′ *and *z ≠ x′*. Based on the assumption of no breakpoint reuse, observing either {*x′, w*} or {*y′, z*} in the derivative genome precludes the existence of {*x′, y′*}, and demonstrates that *A *is open. We call this signature *counterpart-asymmetry *(Figure 1B).

**Definition 2 ***Given a set $\tilde{A}$of experimentally detected adjacencies, A *= {*x, y*} *∈*$\tilde{A}$*has *counterpart-asymmetry *if there exists a breakend w such that w ≠ y′ and *{*x′, w*} *∈ *$\tilde{A}$*or there exists a breakend z such that z ≠ x′ and *{*y′, z*} *∈ *$\tilde{A}$.

The second signature of open adjacencies relies on copy number. We represent copy number as an integer-valued function N on genomic coordinates. Assuming the *k*-break model of rearrangement, each discontinuity in N occurs at a site of breakage and results in a distinct copy number state over each breakend in a counterpart pair. Thus, if a break between nucleotides *n *and *n *+ 1 produces a pair of counterpart breakends: *x *= (*n*, +) and *x′ *= (*n *+ 1*, −*), N(*x*) represents the absolutely copy number state immediately upstream of the break and N(*x′*) the copy number downstream. In addition to the absolute copy number at a breakend, we wish to characterize the change in copy number change *across *a breakend. Thus, we define Δ(*x*) := N(*x′*) *− *N(*x*) where *x′ *is the counterpart of *x*.

In this formulation, breakends flanking deleted regions have negative Δ values. For example, suppose the adjacency *A *= {*x, y*} resulted from a heterozygous (single copy) deletion. Then *x′*, the counterpart of *x*, must lie within the deleted region, meaning N(*x′*) = N(*x*) *− *1 =*⇒ *Δ(*x*) = *−*1. A similar argument implies that Δ(*y*) = *−*1. In this case, the changes in copy number are symmetric at the two breakends of the adjacency. Alternatively, an adjacency *A *= {*x, y*} may exhibit different copy number changes across both its breakends. Such an occurrence is our second signature of open adjacencies called *copy-number asymmetry*, which we define as follows.

**Definition 3 ***Given a set *$\tilde{A}$*of experimentally detected adjacencies, A *= {*x, y*} *∈ *$\tilde{A}$*has *copy-number asymmetry *provided *Δ(*x*) ≠ Δ(*y*).

It is not immediately clear that an adjacency with copy-number asymmetry is necessarily an open adjacency, so we prove the following.

**Proposition 1 ***If an adjacency A has copy-number asymmetry, then it is open*.

*Proof *Suppose that *A *= {*x, y*} is a closed adjacency formed by a *k*-break at some time *t*_0_. This means that the pairs of breakends {*x, x′*} and {*y, y′*} were connected before time *t*_0_, and the pairs {*x, y*}, {*x′, y′*} are connected after time *t*_0_. Since we assume there is no breakpoint reuse, *x *and *y *must have been 'untouched' before time *t*_0_. Thus, N(*x*) = N(*x′*) and N(*y*) = N(*y′*) before *t*_0_. After *t*_0_, these counterpart breakend pairs are no longer fused, meaning their copy numbers can change independently. However, the newly adjacent breakend pairs are now 'locked' to each other and their copy numbers must rise and fall together. For example, once *x *and *y *are adjacent, a copy number decrease over *x *implies a copy number decrease over *y*. Indeed their copy numbers could only change differentially if they were re-broken, violating the assumption that breakpoints are not reused. This implies that in the derivative genome, N(*x′*) *− *N(*x*) = N(*y′*) *− *N(*y*) *⇒ *Δ(*x*) = Δ(*y*), which means closed adjacencies cannot be copy-asymmetric. Conversely, adjacencies with copy-number asymmetry must be open.   □

We emphasize here the importance of analyzing the differences Δ(*x*) and Δ(*y*) in copy number *across *breakends to define copy-number asymmetry rather than absolute copy numbers N(*x*) and N(*y*) *at *breakends. N(*x*) and N(*y*) can be unequal even when the adjacency {*x, y*} is closed, as a change in copy number for either breakend may have occurred *prior *to the formation of the adjacency {*x, y*}. After the formation of the adjacency {*x, y*}, however, copy number changes that affect *x *must also apply to *y *since the two breakends are fused. Thus assuming {*x, y*} is closed, we expect to find Δ(*x*) = Δ(*y*) even when N(*x*) *≠ *N(*y*). Critically, this argument rests on our assumption that there is no breakpoint reuse, since a second break at *x *or *y *(on the originally rearranged chromosome or its homologue) would allow Δ(*x*) and Δ(*y*) to vary independently.

### Counterpart- and copy-number asymmetry cooperate to detect a range of open adjacencies

Since counterpart-asymmetry relies on the presence of counterpart breakends and copy-number asymmetry implicitly relies on their absence, the two signatures in combination can identify a broader set of open adjacencies than each can on its own. This is illustrated in the following two examples.

First, let *A *= {*x, y*} be a closed adjacency. This implies that *x′ *and *y′ *were fused in the *k*-break that created *A*. Clearly, observing the adjacency {*x′, y′*} in the derivative genome would demonstrate that *A *is closed, but what if {*x′, y′*} is not observed? There are two possible explanations: either {*x′, y′*} exists in the derivative genome but was not detected, or the genomic segment containing {*x′, y′*} is deleted. In the latter case, the deletion would have a occurred at the same time as the creation of *A *(i.e. *A *was created by a deletion) or subsequent to the creation of *A*. Since the deletion of an adjacency entails a copy number drop at its constituent breakends and our no breakpoint reuse assumption implies that any subsequent copy number changes would produce coordinated copy number changes across *x *and *y*, we have that Δ(*x*) = Δ(*y*). Thus, *A *would show counterpart symmetry if {*x′, y′*} were retained and copy-number symmetry if it were deleted. In either case, *A *will be considered a closed adjacency according to our definitions (Figure 1A).

Next, suppose *A *= {*x, y*} is an open adjacency. This means that the counterpart *x′ *was fused to a breakend *w ≠ y′*, producing an adjacency {*x′, w*}. Observing the adjacency {*x′, w*} in the derivative genome would demonstrate that *A *is open through counterpart-asymmetry. On the other hand, if the DNA supporting {*x′, w*} were deleted, then there would be a copy number change at *x *(Δ(*x*) *≠ *0). Since *y′ *is not adjacent to *x′ *or *w*, it is unlikely that *y′ *is also deleted at the same time. If we also assume that *y′ *does not experience an independent change in copy number at another time, then we have Δ(*y*) = 0. Under these conditions Δ(*x*) *≠ *Δ(*y*), giving *A *copy-number asymmetry. Therefore, *A *would look open to our signatures if either {*x′, w*} were retained and measured or if {*x′, w*} were deleted and *y′ *were retained (Figure 1B).

### Open adjacency rate

Given a collection of measured adjacencies $\tilde{A}$ and a copy number profile N, we identify the adjacencies that exhibit counterpart-asymmetry or copy-number asymmetry and form a putative set of open adjacencies O *⊂ *$\tilde{A}$. Note that $\tilde{A}$ may represent all measured adjacencies, or a subset of adjacencies that suspected to reflect a chromothripsis-like or chromoplexy-like event. To estimate the proportion of adjacencies in $\tilde{A}$ formed by (*k >*2)-breaks, we define the *open adjacency rate *(OAR)

(1) $$OAR\;\left(\tilde{A},N\right)=:\frac{\left|O\right|}{\left|\tilde{A}\right|}.$$

In real data, not all open adjacencies will display copy-number asymmetry or counterpart-asymmetry. For example, if only a sparse set of adjacencies is detected, then counterparts will be rare. However, those adjacencies which do show either signature can be called open with high-confidence. Hence the total number of adjacencies exhibiting counterpart/copy-number asymmetry bounds the true number of open adjacencies from below. Thus, if there is no experimental error generating false-positive open adjacencies then it follows from Observation 1 that *OAR*($\tilde{A}$, N) *<*$\left|{\tilde{A}}^{k}\right|$/|$\tilde{A}$|.

### Copy-number asymmetry enrichment

For two breakends to be considered counterparts, they must satisfy several criteria, including that they lie close together on the genome. Therefore, in regions that exhibit a dense clustering of breakends it can become difficult to disambiguate breakends that are close because they are counterparts from those that are close due to other factors. Thus, adjacencies which are densely clustered may occasionally appear open due to false positive counterpart breakend calls, artificially enhancing the open adjacency rate. Since adjacency sets representing putative chromothripsis/chromoplexy events are often formed on the basis of breakend clustering [5], it is desirable to develop a measure which ignores the relative positions of breakends and allows one to separate the contribution of breakend clustering from other factors when assessing whether the given adjacencies were formed during a one-off event. We introduce a second measure, *copy-number asymmetry enrichment *(CAE), that imputes the open adjacency rate using only relative copy number changes at adjacent breakends.

Consider an adjacency set $\tilde{A}$ produced by *k*-breaks with *k ≥ *2. Let $\tilde{A}$^2 ^be the set of adjacencies from 2-breaks and $\tilde{A}$*^k ^*be the set from (*k >*2)-breaks, so that $\tilde{A}$ = $\tilde{A}$^2 ^*∪ *$\tilde{A}$*^k^*. Further, let C *⊆ *$\tilde{A}$ denote the subset of copy-number asymmetric adjacencies. We wish to estimate the fraction of adjacencies in $\tilde{A}$ that came from (*k >*2)-breaks using copy-number asymmetry alone; i.e. to estimate |$\tilde{A}$*^k ^|/|*$\tilde{A}$| from |C|. Proposition 1 tells us that |C*| ≤ *$\left|{\tilde{A}}^{k}\right|$. Turning this lower bound into a direct estimate requires quantifying the degree to which $\left|{\tilde{A}}^{k}\right|$ exceeds |C|. This depends critically on the fraction of breakends in $\tilde{A}$ that co-locate with changes in copy number.

Let *p*_Δ _be the fraction of breakends *x *in $\tilde{A}$ such that Δ(*x*) *≠ *0 (i.e. the fraction of breakends co-locating with a change in copy number). To derive an expected relationship between |C*|, p*_Δ _and $\left|{\tilde{A}}^{k}\right|$, we treat the copy number changes Δ(*x*) as random variables and make the following assumptions: (1) For each breakend *x*, Δ(*x*) is always -1 or 0 (deletion or non-deletion); (2) For each adjacency {*x, y*} *∈ *$\tilde{A}$^2^, Δ(*x*) and Δ(*y*) are equal (dependent) and Bernoulli distributed with *P *(Δ(*x*) = Δ(*y*) *≠ *0) = *p*Δ; (3) For each adjacency {*x, y*} *∈ *$\tilde{A}$*^k ^*, Δ(*x*) and Δ(*y*) are independent and Bernoulli distributed with *P *(Δ(*x*) *≠ *0) = *P *(Δ(*y*) *≠ *0) = *p*_Δ_. It follows from these assumptions that ${\tilde{A}}^{2}\cap \text{C}=\emptyset$ and that for an adjacency {*x, y*} *∈ *$\tilde{A}$*^k ^*chosen uniformly at random, *P *({*x, y*} *∈ *C) = *P *(Δ(*x*) *≠ *Δ(*y*)) = *P *(Δ(*x*) = 0, Δ(*y*) = *−*1) + *P *(Δ(*x*) = *−*1, Δ(*y*) = 0) = 2*p*_Δ_(1 *− p*_Δ_). It follows that E(|C|) = 2*p*Δ(1 *− p*_Δ_)$\left|{\tilde{A}}^{k}\right|$, allowing us to approximate $\left|{\tilde{A}}^{k}\right|$ ≈ |C|/(2*p*_Δ_(1 *− p*_Δ_)). Thus, we can estimate (|$\tilde{A}$*^k^|/|*$\tilde{A}$|), the fraction of (*k >*2)-breaks, by the *copy-number asymmetry enrichment *(CAE) ratio, defined as

(2) $$CAE\left(\tilde{A}\right):=\frac{\left|C\right|}{2p_{\Delta }\left(1-p\Delta \right)|\tilde{A}|}.$$

### Detecting open adjacencies in real data

Detecting open adjacencies in real sequencing data requires: (1) a set $\tilde{A}$ of measured adjacencies along with an annotation of the corresponding breakends for membership in counterpart pairs; (2) a copy number profile N across the genome that maps copy number changes to breakends. The procedures we use to collect this data are described below.

We assume that a collection of rearrangements, or structural aberrations, has been identified in the derivative genome by analyzing paired-read or split read data using one of the many algorithms for this purpose [16-18]. The output of these algorithms is a collection V of pairs of breakends {*x, y*} representing novel adjacencies in the derivative genome, where *x *and *y *are oriented genomic coordinates in the reference genome. We form the the adjacency set Ã from V by identifying counterpart breakend pairs {*x, x′*} such that *x, x′ ∈ *V, *x ≤ x′*, and the following criteria are satisfied: (1) *x′ − × ≤ D *for a small integer *D*; (2) *x *has positive orientation and *x′ *has negative orientation; i.e. the pair (*x, x′*) has convergent (+*, −*) orientation; (3){*x, x′*} ∉ V; (4) no other breakends in V lie between *x *and *x′*. In principle, counterpart breakends occupy adjacent nucleotides, so that we expect *x′ − × *= 1, indicating a distance threshold of *D *= 1 in criterion (1) above. However, higher values of *D *may be used in practice since many structural aberration algorithms do not identify breakends to single nucleotide resolution. In addition, counterpart breakends may be separated by a small distance due to microdeletions or "deletion bridges" [4] that occur at rearrangement breakpoints.

One may compute the OAR on the full set of novel adjacencies; i.e. build $\tilde{A}$ from V. Alternatively, one may evaluate a subset of detected adjacencies, for example a spatially clustered set of adjacencies or a collection previously implicated as representing a chromothripsis-like event, by building $\tilde{A}$ from a subset of V. We use the later approach in our analyses below.

To create a copy profile N which maps changes in copy to breakends, we analyze a whole-genome segmentation as follows. First, we match the ends of copy number segments (indicating a change in copy number) to nearby breakends. This is done by creating a breakpoint interval *I *with length *L *around the boundary of each copy number segment For each breakend *x *and breakpoint interval *I*, we declare a match if: (1) *x *lies within *I*; (2) *x *is the only breakend occupying this interval. Since determination of absolute copy number in tumors is challenging due to heterogeneity [19], we assign change in copy values Δ to breakends using a step function: Δ(*x*) = 1 for breakends matched to intervals indicating positive copy change; Δ(*x*) = *−*1 for breakends matched to intervals indicating negative copy change; Δ(*x*) = 0 for breakends without a matched copy change.

## Results

We compute the OAR and CAE on two cancer sequencing datasets: (1) 64 genomes representing seven tumor types from the The Cancer Genome Atlas (TCGA) that were analyzed for chromothripsis by Malhotra, *et al*. [7]; (2) 57 prostate cancer genomes that were analyzed for chromoplexy by Baca, *et al*. [4]. Both studies mapped somatic structural variants and copy number variants, and annotated these variants as representing chromothripsis/chromoplexy or stepwise events. For each dataset, we use the procedures described above to compute the set of observed adjacencies $\tilde{A}$ and copy number profile N from the novel adjacencies V and segmented copy number data reported in the supplemental material of each publication.

### Data processing: adjacency sets and copy number changes

For each dataset we generated a collection of adjacency sets {$\tilde{A}$} to evaluate with our measures, and derived an estimate $\hat{k}\left(\tilde{A}\right)$ of the proportion of adjacencies that were reported to occur by (*k >*2)-breaks. For each TCGA genome, Malhotra *et al*. [7] report a list of observed adjacencies and identify clusters of co-localizing adjacencies which they classify as either "stepwise" or "one-off". The classification was based primarily on number of distinct copy number states observed. We form one adjacency set from each reported cluster, assigning $\hat{k}\left(\tilde{A}\right)=1$ for one-off clusters and $\hat{k}\left(\tilde{A}\right)=0$ for stepwise clusters. We group the adjacencies from each genome not assigned to a cluster by [7] into a "background" adjacency set with $\hat{k}\left(\tilde{A}\right)=0$. We removed all sets containing fewer than 15 adjacencies, leaving 74 adjacency sets. Of these, 8 adjacency sets were classified as one-off and 66 as stepwise. In addition to providing a list of observed adjacencies for each prostate cancer genome, Baca, *et al*. [4] developed and used the ChainFinder algorithm to analyze the prostate cancer adjacencies for chromoplexy and report each chromplexy event as as a "chain" of simultaneously formed adjacencies. Because chromoplexy often spans many chromosomes, we formed adjacency sets containing all measured adjacencies for a genome, and set $\hat{k}\left(\tilde{A}\right)$ to be the proportion of adjacencies with at least one breakend belonging to a chromoplexy chain as reported by [4]. We removed adjacency sets with fewer than 15 adjacencies. The resulting 50 adjacency sets had mean $\hat{k}\left(\tilde{A}\right)$ of 0.501 with standard-deviation 0.24. Further details are included in Additional file 1.

**Figure 2:**
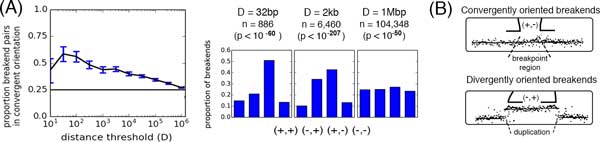
**Summary of counterpart breakend calls**. Breakends are called *counterparts *when they have a convergent (+*, −*) orientation and are close together on the genome. **(A) **(Left) Proportion of all breakends pairs in (+*, −*) orientation (*y*-axis) at varying distance thresholds *D *(*x*-axis), showing excess of convergent pairs at small distances. (Right) Proportion of breakend pairs with each of four possible orientations, at different distance thresholds *D*. The convergent (+*, −*) orientation is enriched at small distances (*p <*10^*−*60 ^for *D *= 32bp, binomial test), suggesting that the breakend pairs are indeed counterparts, and unlikely to result from chance or frequent breakage at closely located sites. **(B) **A mild enrichment of divergent breakend pairs at intermediate distances (e.g. *D *= 2kb) suggests the occurrence of focal duplications resulting from copy-and-paste insertions at breakpoint junctions.

For each adjacency set, we matched breakends into counterpart pairs. To be called counterparts, two breakends must satisfy several criteria including falling within a certain fixed distance *D *(see Methods for further details). We set *D *= 2kb and identified 1,022 counterpart breakend pairs from a total of 11,775 adjacencies reported by both studies. These closely localized pairs are unlikely to have arisen by chance, since the proportion of breakend pairs within distance *D *that display the convergent orientation (+*, −*) is higher than the expected value of 0.25 if orientation pairs were selected uniformly from the four orientations (Figure 2A). This difference is statistically significant (e.g. *p <*10*^−^*^207 ^for *D *= 2kb, binomial test) and peaks when *D *= 32bp, which is less than the insert size of Illumina sequencing, and a reasonable breakpoint localization with multiple supporting read pairs [16]. Surprisingly, the divergent orientation (*−*, +) is also over-represented for small values of *D*. However, this may reflect a high prevalence of templated insertions at translocation junctions (see Figure 2B and Additional file 1).

Next, we created a copy number profie which associates copy number changes to breakends. Supplementary data from both studies reported a total of 41,814 copy number changes across the full set of genomes. We created the copy number profile using the approach described in Methods where the length of breakpoint intervals *L *was set to 10kb. Our approach mapped 6,733 breakends to changes in copy number. It is unlikely that these matches occurred by chance since the matched breakends tended to lie at the centers of their assigned breakpoint intervals (Figure S1B in Additional file 1).

### Open Adjacency Rate (OAR) for cancer genomes

**Figure 3:**
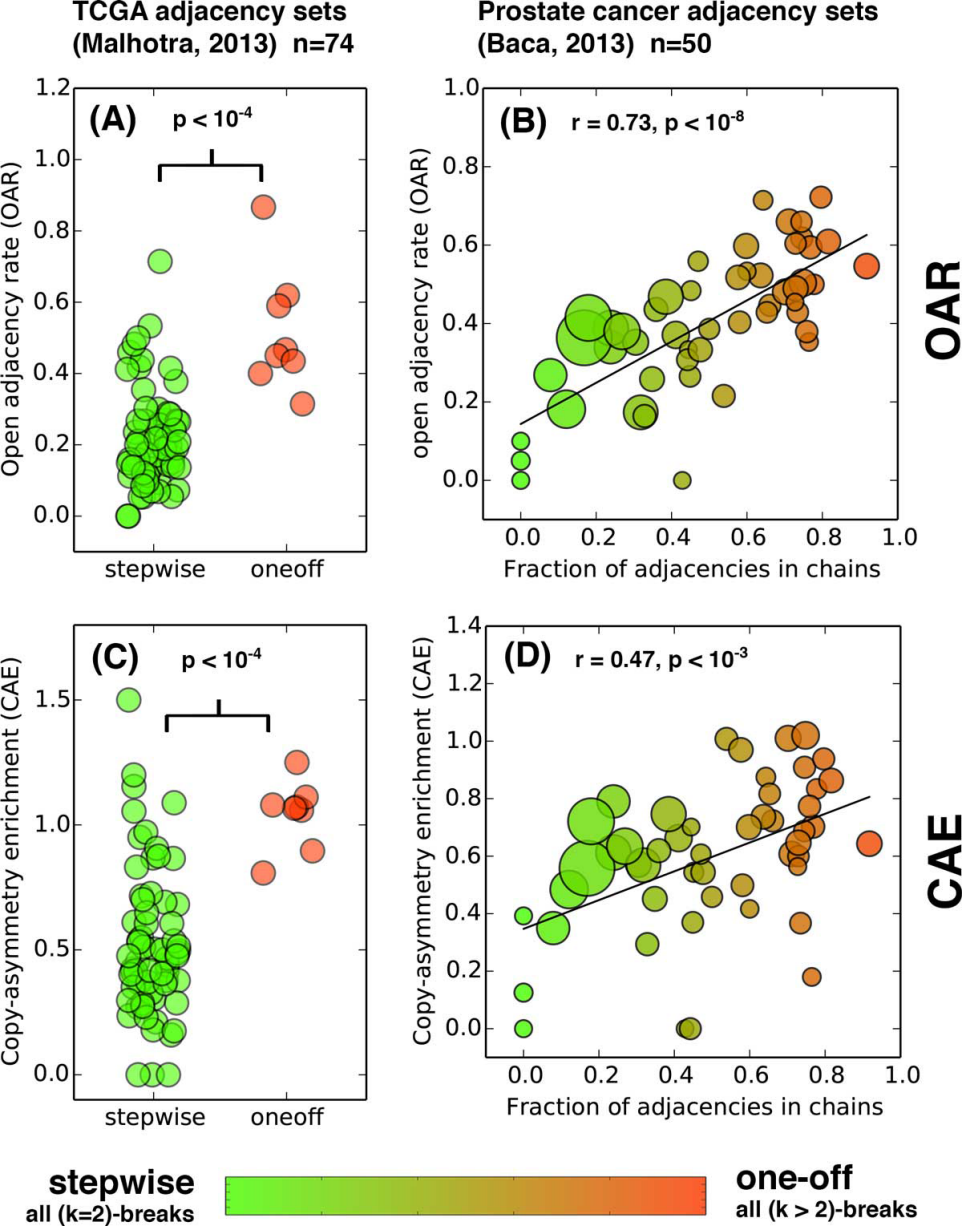
**OAR and CAE of adjacency sets from cancer genomes**. **(A) **Distribution of OAR on 74 adjacency sets from TCGA genomes that were classified as stepwise (all (*k >*2)-breaks) or one-off (all (*k *= 2)-breaks) in [7]. **(B) **Correlation between OAR(Ã) and $\hat{k}\left(\tilde{A}\right)$, the estimated proportion of adjacencies originating in (*k >*2)-breaks, for 50 prostate cancer adjacency sets from [4]. Red dots represent one-off sets and green stepwise are stepwise sets. Dot size indicates number of adjacencies in the set. **(C, D) **The CAE for the corresponding datasets.

**Figure 4:**
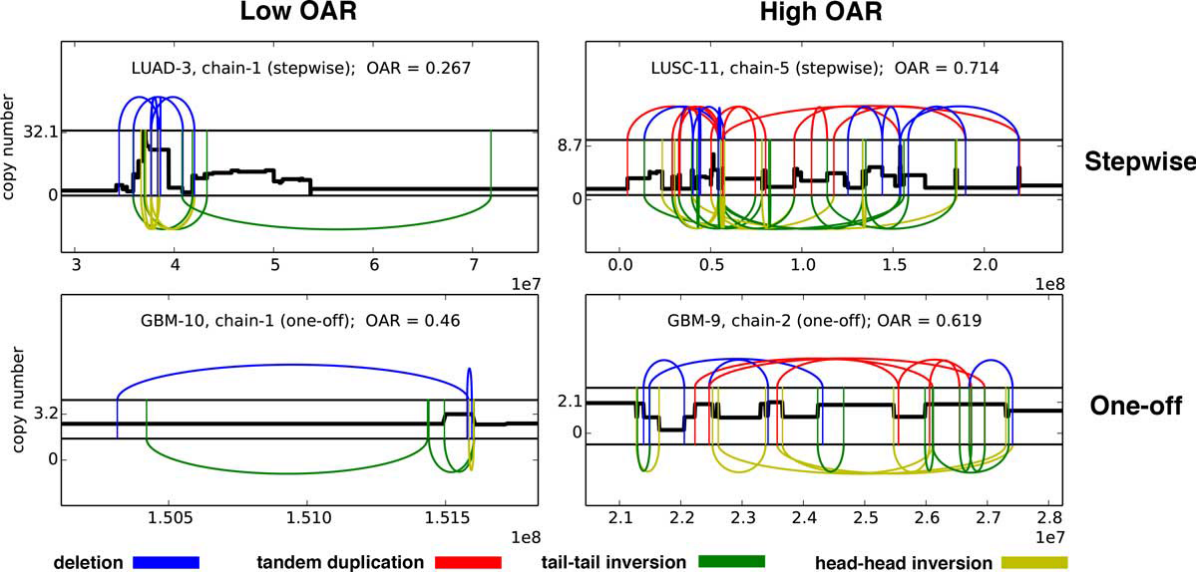
**Examples of agreement/disagreement between OAR and published classifications**. While OAR correlates well with previous classifications of chromothripsis/chromoplexy (Fig. 3), there are several exceptions. (Top right and bottom left) Two examples of differences between OAR and published classifications. (Top left, Bottom right) Two examples of agreement between OAR and published classifications. There are few apparent differences between the examples in each column, supporting the classification of these genomes by the OAR.

We computed the OAR on both sets of cancer genomes. On TCGA genomes, we found that the OAR values for adjacency sets classified as "stepwise" in [7] had significantly lower OAR values (mean OAR = 0.21) than adjacency sets classified as "one-off" (mean OAR = 0.51), *p <*10^*−*4^, Mann-Whitney test (Figure 3A). Both counterpart-asymmetry and copy-number asymmetry contributed to the high OAR values for one-off genomes (Figure S2A in Additional file 1). While our results using the OAR tend to agree with the analysis performed by [7], there are several instances where we obtain differing results. For example, there are four adjacency sets classified as stepwise by [7], but whose OAR scores are within 0.1 of the mean OAR score for other one-off adjacency sets. This indicates that these sets may have been one-off events which were originally mis-classified as stepwise by [7]. To explore this possibility, we visualized these adjacency sets and compared them to stepwise sets with low OAR and one-off sets with high OAR (Figure 4), observing a high similarity with the high OAR one-off sets and supporting our hypothesis that these are actually one-off events.

Next, we computed the OAR for the prostate cancer genomes from [4]. Because the estimates $\hat{k}\left(\tilde{A}\right)$ for these sets were distributed between 0 and 1, we computed the correlation between OAR($\tilde{A}$, N) and $\hat{k}\left(\tilde{A}\right)$ across the adjacency sets. We find that the OAR correlates well with the estimates for $\hat{k}\left(\tilde{A}\right)$ having *r *= 0.73, *p <*10^*−*8^, Pearson test (Figure 3B). For each dataset, both copy-number asymmetry and counterpart asymmetry contributed to the high OAR in adjacency sets with large $\hat{k}\left(\tilde{A}\right)$ (Figure S1 in Additional file 1).

### Copy-number asymmetry enrichment (CAE) on cancer genomes

Since the adjacency sets with $\hat{k}\left(\tilde{A}\right)=1$ among TCGA genomes were identified using clustering of breakends and the estimates $\hat{k}\left(\tilde{A}\right)$ for prostate cancer genomes were assigned based on chains from [4] - which rely on breakend clustering - we expected to find some amount of counterpart asymmetry in these datasets. To remove the contribution of counterpart asymmetry, we computed the CAE on both datasets. On TCGA genomes, we found a clear difference in CAE between adjacency sets classified as "one-off" vs. those classified as "stepwise" (*p <*10^*−*4^), as shown in Figure 3C. On the prostate cancer genomes, we found that the CAE values correlated with $\hat{k}$ (*r *= 0.47, *p <*10^*−*3^, Figure 3D). In addition, the CAE showed significant agreement with the OAR across the collection of all adjacency sets (*r *= 0.67, *p <*10^*−*17^). Overall the CAE predicted (*k >*2)-break prevalence relatively accurately, correlating with previous prediction of chromothripsis/chromoplexy in a manner similar to the full OAR. These results show that copy-number asymmetry can be used to predict open adjacencies (and hence putative (*k >*2)-breaks), providing a measure for detection of simultaneous rearrangements that is independent of measures based on the location of breakends from a set of adjacencies.

## Discussion

The definition of rigorous criteria to distinguish chromothripsis/chromoplexy from stepwise accumulation of rearrangements using DNA sequencing data from a single time point is challenging task [2,4,5,7,11,10,20]. We introduced two measures, the open adjacency rate (OAR) and copy-number asymmetry enrichment (CAE), to quantify the occurrence of simultaneous rearrangements, or *k*-breaks [13] with *k >*2, in the formation of a derivative genome. We showed that the OAR and CAE measures correlate well with previously published analyses [7,4] of chromothripsis/chromoplexy, but that our measures also reveal some potential misclassifications in these studies.

While our results demonstrate that the OAR and CAE are useful measures, they both have limitations. The OAR and CAE are *local *measures that estimate the proportion of (*k >*2)-break adjacencies by considering each adjacency in turn, rather than examining their global configuration. While some information is lost in this approach, robustness to experimental error is gained. Indeed, measures of chromothripsis/chromoplexy that rely solely on the global configuration, such as ChainFinder [4] may be affected by a single missing adjacency. Combining information from global configurations with local measures such as the OAR is therefore an important area for future investigation. In addition, recent studies suggest that chromothripsis/chromoplexy events do not occur in isolation [20]. Thus, flexible measures, such as the OAR and CAE, may be better able to distinguish the available signal of a one-time event from the noise of sequential rearrangements in the same region.

The ability to detect chromothripsis/chromoplexy using OAR, CAE, or related measures is impacted by the extent of intra-tumor heterogeneity within a sample. If a chromothripsis/chromoplexy event exists in only a fraction of cells in the sample, then the power to detect the adjacencies and copy number changes that characterize this event is diminished. Recently developed methods to characterize intra-tumor heterogeneity within a single sample [19,21,22] or new single cell sequencing approaches [23], may provide better data for measures such as OAR.

## Conclusions

We introduce two measures for chromothripsis/chromoplexy, the open adjacency rate (OAR) and copy-number asymmetry enrichment (CAE). We find that these measures correlate well with previously predicted classifications of chromothripsis/chromoplexy on 121 cancer genomes from two different studies, with a few notable exceptions. Visual inspection of the genomes for which OAR makes differing predictions suggest that they have been original mis-classified. Ultimately, *in vivo *or *in vitro *studies of chromothripsis/chromoplexy are necessary to further quantify the causes and prevalence of these events. In the interim, analytical methods to predict *k*-breaks from high-throughput sequencing data will remain useful tools, with the caveat that for some samples such *post hoc *analysis may insufficient to determine reliability whether a chromothripsis/chromplexy event occurred.

## List of abbreviations used

OAR: open adjacency rate; CAE: copy-number asymmetry enrichment; DCJ: double cut and join; DSB: double-stranded breaks; TCGA: The Cancer Genome Atlas

## Competing interests

The authors declare that they have no competing interests.

## Authors' contributions

CW, LO and BJR conceived of the project and wrote the manuscript. CW and LO implemented the measure and performed data analysis.

## Supplementary Material

Additional file 1 **A PDF containing additional details and results not included in the main text**. Click here for file

## Declarations

The publication charges for this article were funded by NIH-NHGRI grant R01HG005690.

This article has been published as part of *BMC Genomics *Volume 15 Supplement 6, 2014: Proceedings of the Twelfth Annual Research in Computational Molecular Biology (RECOMB) Satellite Workshop on Comparative Genomics. The full contents of the supplement are available online at http://www.biomedcentral.com/bmcgenomics/supplements/15/S6.
