# Supplementary material for: Open adjacencies and k-breaks: detecting simultaneous rearrangements in cancer genomes
Source: BMC Genomics. 2014 Oct 17;15(Suppl 6):S4. doi: 10.1186/1471-2164-15-S6-S4 (PMC4239675; doi:10.1186/1471-2164-15-S6-S4)
Supplement: Additional file 1 — A PDF containing additional details and results not included in the main text. [file 1471-2164-15-S6-S4-S1.pdf]

RESEARCH

Supplemental Material: Open adjacencies and k-breaks: detecting simultaneous rearrangements in cancer genomes

Caleb Weinreb[1†], Layla Oesper[1,2†] and Benjamin J Raphael[1,2*]

*Correspondence:
braphael@brown.edu
[1]Center for Computational Molecular Biology, Brown University, Providence, RI, USA
Full list of author information is available at the end of the article
†Equal contributor

Formation of TCGA adjacency sets

For the TCGA genomes, we used the breakpoint clusters identified by Malhotra *et al.* [1] as the basis for our adjacency sets. The chains were produced in a two step process that involved: (1) clustering breakpoints by proximity on the reference genome using a 100kb threshold and (2) chaining together breakpoint clusters spanned by a common adjacency. Malhotra *et al.* further classified the chains as stepwise (i.e. all 2-breaks) or one-off (i.e. chromothripsis). Chains were considered to be one-off when they: (1) displayed three or fewer copy states with at most one copy state greater than four; (2) were not a focal deletion defined as a contiguous region of amplification.

Formation of prostate cancer adjacency sets

For the prostate cancer data, we formed adjacency sets from whole genomes. To estimate $\hat{k}$, we relied on the "chains" identified by Baca *et al.* [2] using a graph-theory based algorithm called ChainFinder. ChainFinder tries to find sets of simultaneously produced adjacencies by linking together breakends that: (1) are connected by an adjacency; (2) are significantly close together on the reference genome according to a custom probabilistic model; (3) bound a focal deletion, meaning there is a drop in copy number across the first breakend and a rise across the second.

In contrast to the spatially clustered chains from [1] which could be classified as either one-off or stepwise, the chains identified by ChainFinder are created on the basis of inferred temporal clustering and are therefore one-off by design. For this

reason, we could not directly translate the chains from [2] into testable adjacency sets, as there would have been no stepwise chains to serve as true-negatives for the OAR. Instead, we set $\hat{k}(\tilde{\mathcal{A}})$ for each genome to be the proportion of adjacencies with at least one breakend belonging to a chain. Since chains are supposed to represent simultaneously formed breakends, the prevalence of chained adjacencies should serve as a proxy for the proportion created in $(k > 2)$-breaks.

Breakend clustering in adjacency sets

As noted in the main manuscript, the original adjacency sets have an implicit dependency on breakend clustering. The 'background' adjacency sets, included with the TCGA data, have a depletion of clustered breakends and were assigned $\hat{k} \equiv 0$. Their inclusion among full collection of adjacency sets boosts the relative level of breakend clustering in the one-off adjacency sets, which were all formed on the basis of spatial clustering. We see this effect when restricting analysis of OAR scores to the 22 non-background chains, for which we find a weaker but still significant segregation between one-off and stepwise sets ($p < 2 \times 10^{-2}$). Similarly the chains from [2] that we use to calculate $\hat{k}(\tilde{\mathcal{A}})$ were originally constructed by the ChainFinder method [2] which uses proximity of breakends to link adjacencies into chains.

Enrichment of divergent oriented pairs

As noted in the main manuscript, we observed that in addition to the convergent orientation $(+,-)$, the divergent orientation $(-,+)$ for complementary breakends was also over-represented for small values of D. This may reflect a high prevalence of templated insertions at translocation junctions (Figure 2B). Templated insertions arise frequently in replicative mechanisms of DNA damage such as FoSTeS/MMBIR [3], which is suspected to play a role in some chromothripsis-like SV clusters [4]. Since these insertions arise though a copy-and-paste mechanism, the corresponding breakends should have high absolute copy number and show nonzero change in copy number ($\Delta(x) > 0$). Supporting this view, we found that the divergently oriented breakend pairs had a higher mean absolute copy number than convergent pairs (2.55 vs. 2.09, $p < 10^{-5}$, Mann-Whitney test) and a higher mean change in copy number (1.4 vs. 0.8, $p < 10^{-3}$).

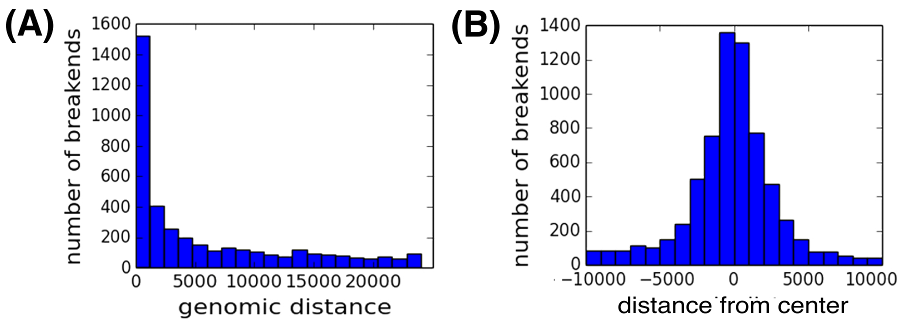

Figure S1 Summary of copy number and complementary breakend class. Breakends were matched to nearby copy number change-intervals if they were the unique breakend lying within a 20kb window concentric with the change-interval. **(A)** Histogram of matched (breakend,chainge-interval) pairs showing deviation of breakend from interval center. The centered distribution suggests that the overlaps did not occur by chance. **(B)** Histogram showing the distance between complementary breakends. To be complementary, breakends had to fall within 25kb and have a convergent $(+,-)$ orientation.

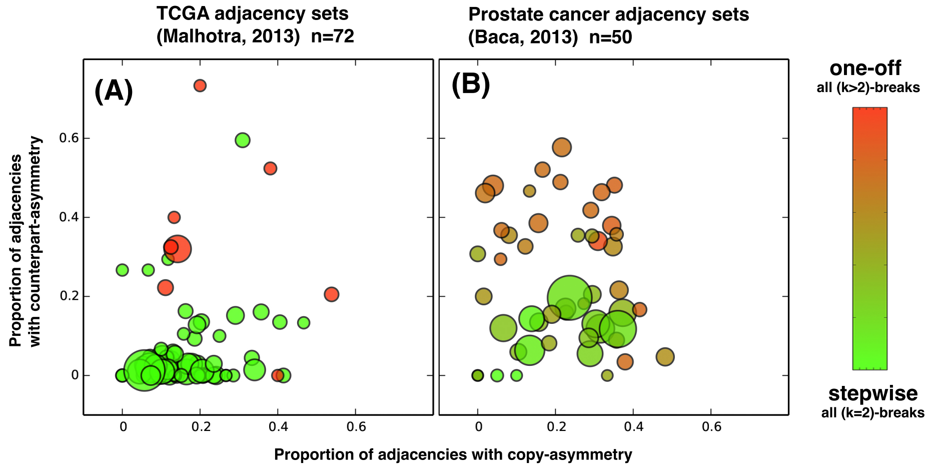

Figure S2 Contribution of copy-asymmetry versus counterpart-asymmetry. The proportion of adjacencies with copy-asymmetry verses counterpart-asymmetry for the **(A)** TCGA adjacency sets **(B)** and the prostate cancer adjacency sets. The color coding of the plots indicates the level of OAR.

Author details

[1]Center for Computational Molecular Biology, Brown University, Providence, RI, USA. [2]Department of Computer Science, Brown University, Providence, RI, USA.

References

1. Malhotra, A., Lindberg, M., Faust, G.G., Leibowitz, M.L., Clark, R.A., Layer, R.M., Quinlan, A.R., Hall, I.M.: Breakpoint profiling of 64 cancer genomes reveals numerous complex rearrangements spawned by homology-independent mechanisms. Genome Research **23**(5), 762–776 (2013). doi:10.1101/gr.143677.112. http://genome.cshlp.org/content/23/5/762.full.pdf+html

2. Baca, S.C., Prandi, D., Lawrence, M.S., Mosquera, J.M., Romanel, A., Drier, Y., Park, K., Kitabayashi, N., MacDonald, T.Y., Ghandi, M., Allen, E.V., Kryukov, G.V., Sboner, A., Theurillat, J.-P., Soong, T.D., Nickerson, E., Auclair, D., Tewari, A., Beltran, H., Onofrio, R.C., Boysen, G., Guiducci, C., Barbieri, C.E., Cibulskis, K., Sivachenko, A., Carter, S.L., Saksena, G., Voet, D., Ramos, A.H., Winckler, W., *et al.*: Punctuated evolution of prostate cancer genomes. Cell **153**(3), 666–677 (2013). doi:10.1016/j.cell.2013.03.021
3. Zhang, F., Khajavi, M., Connolly, A.M., Towne, C.F., Batish, S.D., Lupski, J.R.: The dna replication fostes/mmbir mechanism can generate genomic, genic and exonic complex rearrangements in humans. Nat Genet **41**(7), 849–853 (2009)
4. Liu, P., Erez, A., Nagamani, S.C.S., Dhar, S.U., Kołodziejska, K.E., Dharmadhikari, A.V., Cooper, M.L., Wiszniewska, J., Zhang, F., Withers, M.A., Bacino, C.A., Campos-Acevedo, L.D., Delgado, M.R., Freedenberg, D., Garnica, A., Grebe, T.A., Hernández-Almaguer, D., Immken, L., Lalani, S.R., McLean, S.D., Northrup, H., Scaglia, F., Strathearn, L., Trapane, P., Kang, S.-H.L., Patel, A., Cheung, S.W., Hastings, P.J., Stankiewicz, P., Lupski, J.R., Bi, W.: Chromosome catastrophes involve replication mechanisms generating complex genomic rearrangements. Cell **146**(6), 889–903 (2011). doi:10.1016/j.cell.2011.07.042
